# Head and Neck Surgical Oncology Choosing Wisely Campaign: imaging for patients with hoarseness, fine needle aspiration for neck mass, and ultrasound for odynophagia

**DOI:** 10.1186/s40463-017-0251-x

**Published:** 2018-01-08

**Authors:** Antoine Eskander, Eric Monteiro, Dan O’Connell, S. Mark Taylor, The Canadian Association of Head and Neck Surgical Oncology (CAHNSO)

**Affiliations:** 1 Department of Otolaryngology - Head & Neck Surgery, Sunnybrook Health Sciences Centre and the Odette Cancer Center, Michael Garron Hospital, University of Toronto, 2075 Bayview Avenue, Suite M1-102, Toronto, ON M4N 3M5 Canada; 2 Department of Otolaryngology - Head & Neck Surgery, Mount Sinai Hospital, University of Toronto, Toronto, ON Canada; 3 Division of Otolaryngology - Head & Neck Surgery, University of Alberta, Edmonton, AB Canada; 4 Division of Otolaryngology - Head & Neck Surgery, Dalhousie University, Halifax, NS Canada

**Keywords:** Head and neck surgical oncology, Choosing Wisely Canada, Communication, Medical decision making, Health care delivery

## Abstract

Choosing Wisely Canada, is a campaign designed to raise awareness regarding inappropriate or unnecessary tests and treatments. The Canadian Society of Otolaryngology - Head & Neck Surgery and the Canadian Association of Head and Neck Surgical Oncologists developed a Choosing Wisely Canada list to help promote high quality care for patients presenting with disorders of the head and neck: (1) Don’t order imaging - computer tomography (CT) or magnetic resonance imaging (MRI) - as the initial investigation for patients presenting with a chief complaint of hoarseness, (2) Don’t perform an open biopsy or excision of a neck mass without having first considered a fine needle aspiration (FNA) biopsy and, (3) Don’t order neck ultrasound to investigate odynophagia (discomfort or pain with swallowing) or globus sensation.

## Introduction

Choosing Wisely Canada, is a campaign designed to raise awareness regarding inappropriate or unnecessary tests and treatments. The recommendations which are marketed in medical journals, websites and in physician offices is designed to create a discourse between physicians and their patients regarding smart and effective choices leading to high quality care.

The Canadian Society of Otolaryngology-Head & Neck Surgery (CSOHNS) is a proud partner of the Choosing Wisely Canada campaign. CSOHNS is an association that helps to serve the Canadian Otolaryngology-Head & Neck Surgery community. It is composed almost exclusively of otolaryngologists-head & neck surgeons and those training in the specialty. CSOHNS is dedicated to improving patient care through the support of education, the promotion of research, the dissemination of information, the scientific advancement of the Society, and the maintenance of high professional and ethical standards. The CSOHNS has already developed its first list through the Otology & Neurotology subspecialty group of the society.

Through the CSOHNS, the Canadian Association of Head and Neck Surgical Oncologists (CAHNSO) has spawned as a subspecialty interest group. It is now an independent inclusive group dedicated to improving the quality of head and neck cancer care and education throughout Canada. Its mission is to help promote excellence in the surgical treatment of head and neck cancers and other diseases, and to provide educational resources, help enable research and strive to advance the sub-specialty of Head and Neck Surgery in Canada.

It is therefore natural that the CAHNSO develop a Choosing Wisely Canada list to help promote high quality care for patients presenting with disorders of the head and neck. The list is designed for any physician assessing a patient with a disorder of the head and neck and is designed to avoid inappropriate and unnecessary tests and treatment.

## Methods

This list was created by the CAHNSO subspecialty group of the CSOHNS. A set of guiding principles for the list were adapted and modified from the Choosing Wisely Canada website, [1] to be adopted by the CAHNSO group in the development of the list (Table 1). Members of the group, considered to be national leaders within the subspecialty, created a list of recommendations for unnecessary tests and treatments using these principles. Care was made not to overlap with previous Choosing Wisely Canada or American lists and a thorough search (choosingwisely.org) was used to exclude repeat items. The American Academic of Otolaryngology - Head and Neck Surgery Choosing Wisely Campaigns list was also reviewed to avoid duplicate recommendations.

**Table 1:** Principles guiding CAHNSO Choosing Wisely Canada list*

Common disease process, investigation or test
Within the scope of otolaryngology - head and neck surgery, specifically within head and neck oncology, but recommendations are not limited to this subspecialty and may be helpful for all physicians seeing these patients
The inappropriate or excessive use of health care resource is not helpful to patients or may expose patients to harm or stress
Each list item has to be well supported by evidence

*Adapted and modified from Choosing Wisely Canada [1]

A working group of four members from across Canada was formed to refine the list. The refined list was then presented to the group at the 70th Annual Meeting in Charlottetown, Prince Edward Island (June 2016). Recommendations from the group were incorporated to refine the list and wording of the recommendations. A literature review was undertaken for each list item and is included in this manuscript. The final list was ratified by the CAHNSO executive and members at the 71st Annual CSOHNS Meeting in Saskatoon, Saskatchewan (June 2017).

All authors were involved in the authoring of all of the recommendations. There was unanimous agreement amongst members of the CAHNSO on the final list while contributions in the form of suggestinos, revisions and approval were sought out throughout the process iteratively. These recommendations were heavily edited by Choosing Wisely Canada for both content and format. Furthermore, this submission is based on expert opinion with assessment of the best literature, and is therefore submitted as a letter to the editor.

## Results

### Don’t order imaging - computer tomography (CT) or magnetic resonance imaging (MRI) - as the initial investigation for patients presenting with a chief complaint of hoarseness

Many patients presenting with hoarseness do not have an underlying head and neck malignancy. Hence, ordering imaging initially is not often helpful in making a diagnosis [2]. Persistent hoarseness, lasting greater than 6 weeks, can be one of the first signs of malignancy of the larynx or voice box. This is particularly true in current or ex-smokers and individuals with a current or previous history of alcohol abuse. Laryngoscopy as part of a thorough physical examination is the best initial investigation of persistent hoarseness [3]. If the laryngoscopy demonstrates a vocal cord paralysis or a mass/lesion of the larynx, imaging to further evaluate is evidence-based [4].

When patients are assessed urgently for suspected head and neck cancer, only 3–12% have evidence of a head and neck malignancy [3, 5]. Furthermore, since many patients presenting with persistent hoarseness do not have an underlying head and neck malignancy, ordering imaging reflexively without first visualizing the larynx, is inappropriate, adds costs, and does not help with achieving a diagnosis [2].

### Don’t perform an open biopsy or excision of a neck mass without having first considered a fine needle aspiration (FNA) biopsy

Neck masses may arise from malignant and non-malignant processes (infectious, congenital, traumatic, and benign). An otherwise asymptomatic neck mass may be the only presenting feature of a head and neck malignancy (including squamous cell carcinoma of the mucosa of the upper aerodigestive tract, thyroid, salivary gland and/or other cancers). There is strong evidence that suggests that neck masses in adults should be treated as malignant until proven otherwise [6, 7]. Tissue or cytologic confirmation is imperative to establishing a diagnosis and initiating the appropriate treatment pathway for head and neck cancer.

A fine needle aspiration biopsy (FNA) is the gold standard for initial work up for a neck mass and has numerous advantages over an open neck biopsy [6]. FNA holds less risk and avoids the chance of seeding cancer cells in the neck and making subsequent treatment of a confirmed malignancy more challenging [8]. It is also inexpensive, quickly obtained without a general anaesthetic, and can be performed with or without the use of imaging to assist with the placement of the needle depending on the location of the neck mass, particularly if it is partially cystic or near vital structures. Open neck biopsies should only be considered for a neck mass if the result of an FNA biopsy is non-diagnostic and no primary carcinoma is identified upon a complete head and neck examination. If there is a strong suspicion of lymphoma (previous history of lymphoma, night sweats, weight loss, wide spread lymphadenopathy) an open or core biopsy can be considered in lieu of a FNA [9].

### Don’t order neck ultrasound to investigate odynophagia (discomfort or pain with swallowing) or globus sensation

Odynophagia and globus sensation are common symptoms and the differential diagnosis can be extensive, including inflammatory, infectious, neoplastic, autoimmune and traumatic causes. Globus can, in certain circumstances, be further aggravated by a patients’ lifestyle (smoking, caffeine consumptions, poor sleep hygiene, and poor hydration). Odynophagia and globus sensation are infrequently due to an underlying neck mass, and if so, the underlying lesion is usually quite apparent on physical examination. Neck or thyroid ultrasonography ordered to investigate patients with odynophagia and globus sensation are more likely to detect other entities such as benign thyroid nodules, rather than confirming a diagnosis that explains the patient’s symptoms and can lead to a cascade of other unnecessary tests that can be harmful to patients [10, 11]. Unfortunately, using tests to exclude conditions, can sometimes identify other diseases such as thyroid nodules, leading to further testing such as an FNA or repeat ultrasounds and in some cases treatment in the form of a thyroidectomy that may be unnecessary or harmful to patients [10, 11].

Between 1993 and 2004, there was an increasing rate of yearly neck ultrasounds performed in the province of Ontario [10, 11]. Similarly, the rate of new thyroid cancer cases in the province closely mirrored the increasing rate of neck ultrasonography [10, 11]. The increase in thyroid cancer incidence was predominately for tumours < 2 cm, while the incidence of larger tumours (2–4 cm) remained stable [12]. This is an example where overuse of tests, such as neck ultrasonography, can lead to unnecessary, and potentially harmful treatments for patients. Furthermore, attributing odynophagia to an incidental ultrasound detected thyroid nodule may prevent appropriate examination that may lead to the cause of the symptoms. Thyroidectomy, even in patients with nodules, does not resolve symptoms of odynophagia or globus, unless the mass is otherwise obvious on physical examination with compressive symptoms (inability to swallow solids, liquids or both, or difficulty breathing, typically worse when lying down). Therefore, imaging patients presenting with globus or odynophagia with neck ultrasonography should be avoided, unless the clinical examination reveals any lesions of concern.

## Discussion

These recommendations are not intended for use in making decisions regarding payment by insurers nor should they be used to establish coverage decisions. These lists are meant to stimulate communication between patients and clinicians regarding the appropriateness for a test or treatment and to better understand its role in the management of a suspected disease or diagnosis. These are not intended to be “laws” or rules. Each patient and clinicians should use the specific situation to determine what is best for the patient.

## Abbreviations

CAHNSO: Canadian Association of Head and Neck Surgical Oncologists
CSOHNS: Canadian Society of Otolaryngology-Head & Neck Surgery
FNA: Fine Needle Aspiration Biopsy
